# An Atypical Presentation of Soft Tissue Metastasis in a Patient With Lung Cancer

**DOI:** 10.7759/cureus.16294

**Published:** 2021-07-10

**Authors:** Erika Tvedten, Zachary Deak, Beth Schwartz, Ashlee Rice

**Affiliations:** 1 General Surgery, Detroit Medical Center Sinai-Grace Hospital, Detroit, USA

**Keywords:** case report, adenocarcinoma of unknown origin, subcutaneous metastasis, clinical practice guideline, non-small-cell lung cancer

## Abstract

Soft tissue metastasis in patients with lung cancer is infrequently reported in the literature. Primary lung carcinomas have been shown to exhibit evidence of metastasis to soft tissue in 2.3% of cases. A 75-year-old Caucasian female presented with clinical signs of anemia and the appearance of several soft tissue masses on her back. The patient was a former smoker with a 20-pack-year history. Further workup revealed a diagnosis of adenocarcinoma of the lung with soft tissue metastasis. Recognizing the early signs of metastasis is crucial to providing the patient the best treatment available, and the importance of a thorough physical examination cannot be emphasized enough.

## Introduction

The incidence of lung cancer in the United States has slowly declined over the past few decades. However, as of 2018, lung cancer remains the second most common cancer diagnosed and the most common cause of death from cancer in both sexes [1]. Adenocarcinoma of the lung, a type of non-small-cell lung cancer (NSCLC), is the most common type of lung cancer in smokers and non-smokers, making up 40% of all lung cancers [2]. Unlike other primary cancers, lung cancer has been found to metastasize to all organ systems, and an estimated 50% of patients have metastatic lesions at the time they are diagnosed. In lung cancer patients with end-stage disease, pathology reports have shown evidence of metastases in 93% of cases, with the most common locations being the liver, adrenal glands, bone, kidney, and brain [3].

Although lung metastases to several organ systems are well reported, the literature infrequently recognizes cases of lung metastases to soft tissue including skeletal muscle, subcutaneous tissue, and skin. From the limited available literature, only 2.3% of primary lung carcinomas have been shown to exhibit evidence of metastasis to soft tissue [3].

Guidelines established in 2013 by the United States Preventive Services Task Force (USPSTF) recommend that patients at high risk for lung cancer, such as former heavy smokers, undergo annual screening with low-dose computed tomography (CT) [4]. These screening guidelines are problematic for traditionally lower-risk patients who do not meet the criteria of a 30-pack-year history for annual screening. As a consequence, clinicians must rely heavily on physical examinations, detailed histories, and clinical suspicion for prompt detection. In the early disease stages, patients with adenocarcinoma of the lung are usually asymptomatic and have an unfavorable probability of early diagnosis [2]. Recognizing soft tissue metastasis in patients with lung cancer may lead to earlier detection as well as provide the advantage of a more accessible biopsy.

## Case presentation

A 75-year-old Caucasian female presented to the emergency department with dark-colored stools and generalized weakness for three weeks. The patient also reported concurrent bloating, epigastric pain, and abdominal tenderness. She denied fever, chills, shortness of breath, chest pain, hematemesis, or hematochezia. At a prior office visit with her primary care physician, her hemoglobin concentration had dropped from a baseline of 11 g/dL to 8 g/dL within one week. An in-office fecal occult blood test was negative for blood in her stool. Notably, over the last few months, the patient noticed multiple soft tissue masses on her abdominal wall and right lower back.

The patient had a history of coronary artery disease with a myocardial infarction in 2011 which necessitated percutaneous transluminal coronary angioplasty and stent placement. She underwent a hysterectomy with bilateral oophorectomy 30 years ago, though she denied a history of ovarian and uterine cancer. Her most recent colonoscopy was in 2012 and showed no polyps, metastasis, or areas of bleeding. She quit smoking in 1986 after a 20-pack-year history. Her family history was significant for a brother with unspecified brain cancer; however, no family history of other malignancies was noted. On physical examination, tender soft tissue masses were noted in the right mid-back, periumbilical area, and right chest wall/superior breast.

A contrast CT of the thorax was performed which showed a left lower lobe pulmonary embolus and a spiculated nodule within the right upper lobe. Additionally, scattered solid and ground-glass nodules were present which raised concern for metastasis, as well as anterior lobular septal thickening concerning for lymphangitic metastasis. Moreover, a T10 vertebral body lytic expansile lesion, as well as soft tissue masses throughout both breasts and the anterior abdominal wall, were appreciated, furthering concern for metastasis. A contrast CT of the abdomen and pelvis revealed a cystic lesion in the posterior costophrenic angle measuring 4.5 cm, a 2.4 cm hypodense liver lesion, a 1.7 cm necrotic lesion near the spleen, and a 2.2 cm abdominal wall necrotic mass. In addition, multiple soft tissue nodules were identified throughout the abdominal wall, mesentery, and peritoneum, raising concern for peritoneal carcinomatosis. Splenomegaly and a non-occlusive thrombus in the suprarenal aorta were also appreciated, and a contrast CT of the head showed no metastasis to the brain.

**Figure 1:**
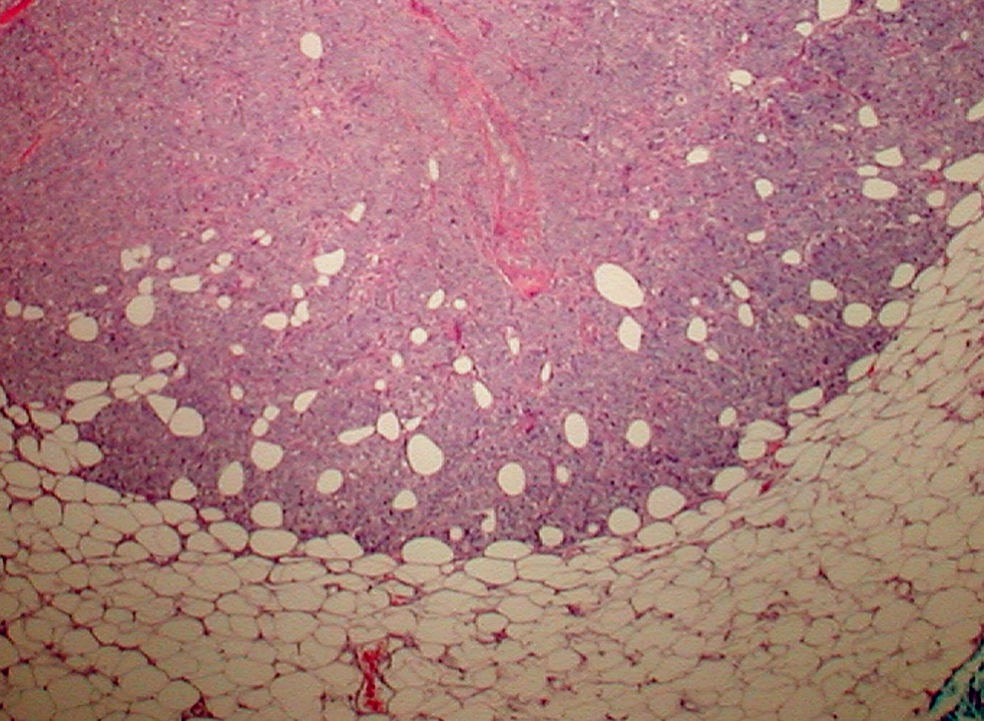
Tumor mass infiltrating the subcutaneous adipose tissue. Hematoxylin and eosin stain, 100×

**Figure 2:**
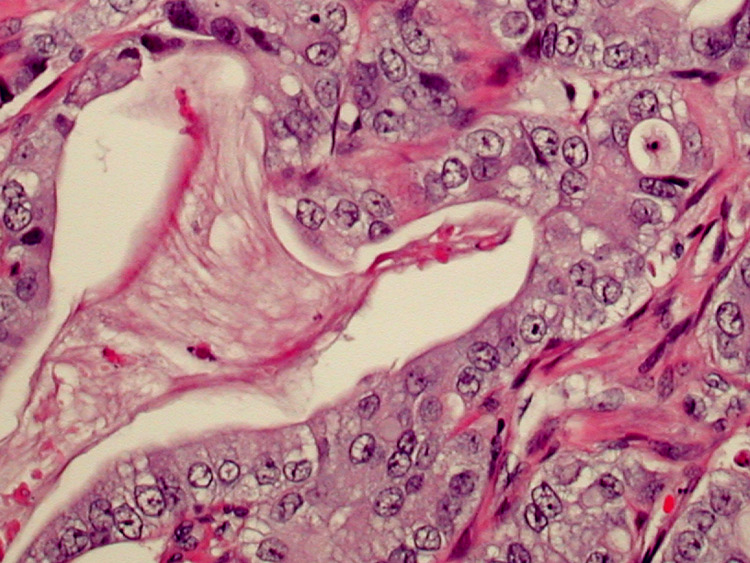
Features of adenocarcinoma can be seen, characterized by moderately pleomorphic tumor cells lining glandular spaces. Hematoxylin and eosin stain, 400×

**Figure 3:**
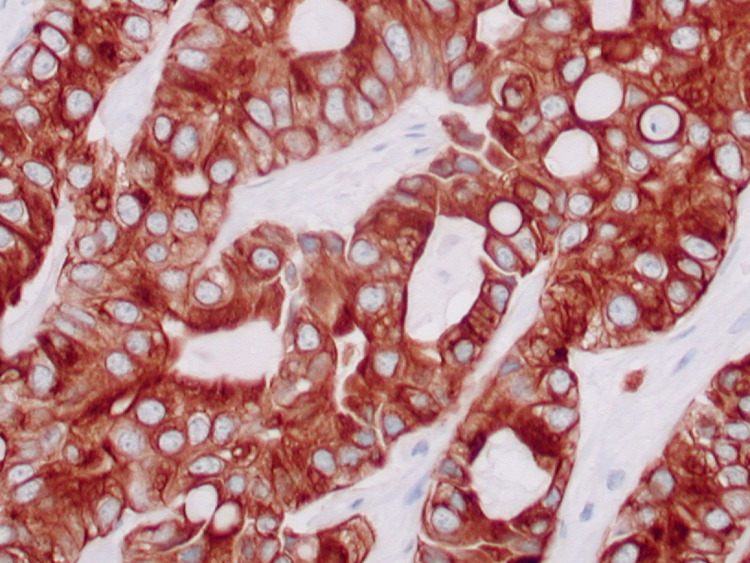
Cytokeratin 7 immunohistochemistry with a strong and diffuse brown cytoplasmic reaction. Immunohistochemical stain, 400×

An esophagogastroduodenoscopy (EGD) with biopsy of the gastric antrum did not identify any pathology that would explain the patient’s anemia. Subsequently, a fine needle core biopsy of a right mid-back mass showed adipose tissue that was extensively replaced by the firm, gray-tan tissue. Histologic sections confirmed the presence of a tumor that was seen infiltrating adipose tissue (Figure 1). The tumor was arranged in small nests and cords with rare signet cells, consistent with moderately differentiated adenocarcinoma. The tumor cells were round to oval with pleomorphic vesicular nuclei containing prominent nucleoli (Figure 2). A panel of immunohistochemistry (IHC) markers was performed and the tumor cells were positive for cytokeratin 7 (CK7) and negative for CK20, thyroid transcription factor 1 (TTF-1), Napsin, GATA binding protein 3, pair box 8 (PAX8), caudal-type homeobox 2 (CDX2), and special AT-rich sequence-binding protein 2 (SATB2) (Figure 3), suggesting that the lungs were the primary origin of the metastatic adenocarcinoma.

The patient was discharged following the correction of anemia. She reported for outpatient follow-up with hematology-oncology when the decision was made to begin chemotherapy for stage IV adenocarcinoma of the lung, the most likely primary site at this time. Her chemotherapy regimen included carboplatin, pemetrexed, and pembrolizumab. Iron studies revealed her anemia to be likely due to chronic disease. Tumor markers were checked including alpha-fetoprotein, carbohydrate antigen 19-9, cancer antigen 125, and carcinoembryonic antigen (CEA), which was elevated at 61 ng/mL. The patient was instructed to maintain regular follow-up with hematology-oncology for chemotherapy and transfusions if necessary.

## Discussion

A 75-year-old female with several soft tissue masses was diagnosed with adenocarcinoma of the lung following fine needle biopsies revealing metastatic tissue. IHC staining following biopsy of the lower back mass was an important tool in the diagnosis of lung adenocarcinoma with subcutaneous metastasis and helped determine the next steps in management. The right-back mass stained positive for CK7 and negative for CK20. CK7 and CK20 are important tumor markers that help differentiate primary lung adenocarcinoma from colorectal adenocarcinoma. Notably, the CK7+/CK20- pattern is found in 96% of primary and 95% of metastatic lung adenocarcinomas, and the CK7-/CK20+ pattern is found in 100% of primary and 88% of metastatic colorectal adenocarcinoma. In 95% of the cases, the CK7 and CK20 staining pattern characterized and differentiated between lung and colorectal adenocarcinoma [5]. For this patient, a CK7+/CK20- staining pattern suggests metastasis from the lung rather than the GI tract. This conclusion is also supported by the negative EGD biopsy. However, it is important to recognize that the presence of lower GI adenocarcinomas cannot be completely excluded with CK7 and CK20 staining patterns [5]. For this patient, a colonoscopy was not performed as histologic findings indicated the primary tumor to likely be from the lung.

TTF-1 is a lung marker that can help differentiate primary lung adenocarcinoma from metastatic lung carcinomas. Although 80% of primary lung adenocarcinomas showed a Napsin A+/TTF-1+ pattern, approximately 20% of primary lung adenocarcinomas stain TTF-1-. Napsin A has a slightly higher sensitivity in detecting primary lung adenocarcinoma. Thus, a positive TTF-1 stain can help rule in lung adenocarcinoma, but a negative TTF-1 stain does not rule out lung adenocarcinoma as the primary source. It is important to note that TTF-1 may also be positive in adenocarcinomas arising in different organs including the colon, endometrium, endocervix, and ovaries [6]. PAX8 is expected to be negative in any primary lung carcinoma [7]. Negative staining of SATB2, a tumor marker that helps differentiate colorectal adenocarcinoma specifically from other adenocarcinomas, and CDX2, a tumor marker that helps to differentiate adenocarcinoma of intestinal origin from other adenocarcinomas, also helps support the exclusion of colon adenocarcinoma as the primary source [8,9].

Based on the IHC findings, it can be inferred that the patient’s primary malignancy origin was the lung. Additionally, the patient’s CEA was elevated at 61 ng/mL. A normal CEA is less than 3 ng/mL. CEA can be a useful prognostic indicator in NSCLC, although as with other tumor markers, it is not useful for screening [10]. At this point, the hematology-oncology team made the diagnosis of stage IV adenocarcinoma of the lung and began chemotherapy treatment.

A paramount lesson that can be highlighted in this particular case is the importance of performing a thorough physical examination including looking at all areas of the skin. Although the patient might not present to the clinic with worries about skin nodules when performing a thorough examination, the mass would be found and further questioning could determine how long the patient had noticed the mass and the rate of growth to determine further investigations. Detection of soft tissue metastasis may have prognostic implications and provide more accessible biopsy sites [11]. This can ultimately help avoid invasive procedures when internal neoplasms are suspected.

Although this an atypical manifestation and finding of NSCLC, healthcare providers may start to see these cutaneous metastatic locations more frequently with patients living longer and more patients achieving remission. As we provide care for patients with significant smoking history and other risk factors for primary lung malignancies, we must maintain a high index of suspicion for cutaneous metastases [12]. Other reports on similar cases suggest a poor prognosis when cutaneous metastases are present [13,14].

As of March 2021, new lung cancer guidelines from the USPSTF were updated and changed the criteria for heavy smoking history from 30-pack-year smoking history to include patients with a minimum of 20-pack-year smoking history [15,16]. The new guidelines recommend annual screening for patients with a heavy smoking history, 20-pack-years or more, who currently smoke or quit within the past 15 years, and who are between 55 and 80 years old [15,16]. The patient would not have fallen into the population recommended for annual low-dose CT screening.

## Conclusions

This case of soft tissue metastasis in lung cancer, although rare, emphasizes the importance of a thorough physical examination, as superficial clues are useful in the diagnosis of various diseases. Though guidelines are updated periodically to best serve population health, patients who do not meet the screening criteria require careful evaluation for the disease. This patient did not meet the lung cancer screening criteria established in 2013 and presented with end-stage disease. While more research in this area is needed, cases such as this may prove useful in ensuring the successful screening of patients in the future.
